# A Retrospective Audit of Healthcare-Associated Infections in Patients Who Are Optimised for Discharge at a Tertiary Medical Centre

**DOI:** 10.7759/cureus.105026

**Published:** 2026-03-11

**Authors:** Christopher Barrett, Harish Shankar Kumar, Helen Singleton

**Affiliations:** 1 Medicine, Leeds Teaching Hospitals NHS Trust, Leeds, GBR; 2 Respiratory Medicine, Leeds Teaching Hospitals NHS Trust, Leeds, GBR

**Keywords:** discharge, hcai, hospital, nhs, social care

## Abstract

Background

This audit aimed to identify patients who developed a healthcare-associated infection (HCAI) after being deemed medically optimised for discharge and to then recognise the common patterns of HCAIs alongside the reasons for delays in discharges.

Methodology

This audit used a retrospective observational design. The audit was conducted in a general medical ward in an NHS England tertiary medical centre between December 2023 and January 2024. The study had a sample size of 93 patients who had been deemed medically optimised for discharge (MOFD) in a general medical ward but had not yet been discharged. The main outcome measures of the audit were the type and period prevalence of HCAI and the reasons for delay in discharge.

Results

In total, 93 patients were determined to be MOFD, with 11 (12%) suffering HCAI. The study identified COVID-19 as the most prevalent HCAI, accounting for four (36.4%) cases, followed by three (27.3%) cases of urinary catheter-related infections, two (18.2%) cases of hospital-acquired pneumonia not related to COVID-19, and two (18.2%) cases of other infections. The study also identified that delays in discharge for patients were mainly due to waits for social care. Overall, 64% (seven patients) of patients had a delay in discharge because of delays in social care, followed by 36% (four patients) due to ongoing therapy needs.

Conclusions

This audit links delays in social care to adverse health outcomes in hospitals. Immediate measures are needed to strengthen social care provisions within hospitals to improve health care outcomes in an increasingly aging population.

## Introduction

The National Institute for Health and Care Excellence (NICE) defines healthcare-associated infections (HCAIs) as any infection contracted: (A) as a direct result of treatment in, or contact with, a health or social care setting; (B) as a result of healthcare delivered in the community; and (C) outside a healthcare setting (for example, in the community) and brought in by patients, staff, or visitors and transmitted to others (for example, norovirus) [1].

The Health Protection Agency (HPA) conducted a study in 2012 (updated in 2016) [2], the Point Prevalence Survey, England, which identified the prevalence and incidence of HCAIs in hospitals in England. The survey revealed that 6.4% of inpatients in acute care hospitals in 2011 had an HCAI. The six most common types of HCAIs, which accounted for more than 80% of all HCAIs, were pneumonia and other respiratory infections (22.8%), urinary tract infections (17.2%), surgical site infections (15.7%), clinical sepsis (10.5%), gastrointestinal infections (8.8%), and bloodstream infections (7.3%).

The World Health Organization (WHO) quoted the European Centre for Disease Prevention and Control (ECDC) in their surveillance of HCAIs at national and facility levels [3], which estimated that the burden of the six most frequent HCAIs was calculated to be twice the burden of 32 other infectious diseases altogether in terms of disability and premature mortality.

HCAIs adversely impact health outcomes and have been shown to significantly impact health economics and increase the financial burden on the healthcare system [4]. NICE Public Health Guidance 36 [5] stresses the importance of reducing inpatient hospital stays and focus of infection prevention and control to reduce the risk and incidence of HCAIs.

## Materials and methods

This was a retrospective observational study focusing on collecting data from general internal medicine wards at St James’s University Hospital, Leeds, United Kingdom. St James’s University Hospital serves as a major tertiary medical centre within NHS England. The data were collected in the months of December 2023 and January 2024.

The sample size focused on patients who were deemed medically optimised for discharge (MOFD) but were not physically discharged from the wards due to a variety of reasons that were also recorded. MOFD date/time referred to the first documented MOFD entry; time-at-risk began at MOFD timestamp.

We reviewed all admissions to the general internal medicine wards and identified patients who had completed treatment for their primary presenting complaint. Patients were eligible for inclusion regardless of the nature of their initial presentation, provided that their acute issue had been fully managed and they had been judged MOFD by the responsible clinical team. As part of the exclusion criteria, we excluded patients who remained under active treatment for their primary presenting complaint. Applying these criteria, 93 patients were deemed eligible for inclusion in the study.

With this data, further analysis focused on the prevalence of HCAIs, the type of infection, and the reasons behind the delay in discharge. The data were then compared with the Point Prevalence Survey conducted by the HPA [2].

The primary outcome of this study was the incidence of HCAIs among patients who were declared MOFD but experienced a delay in discharge. The secondary outcomes included the frequency of these discharge delays along with the underlying reasons for these delays.

The study focused exclusively on general medical patients; therefore, it does not account for HCAIs seen on surgical wards or infections which may result from surgical interventions. The study also only focused on adult patients, thus also excluding the infections which are seen in the paediatric population.

## Results

**Figure 1:**
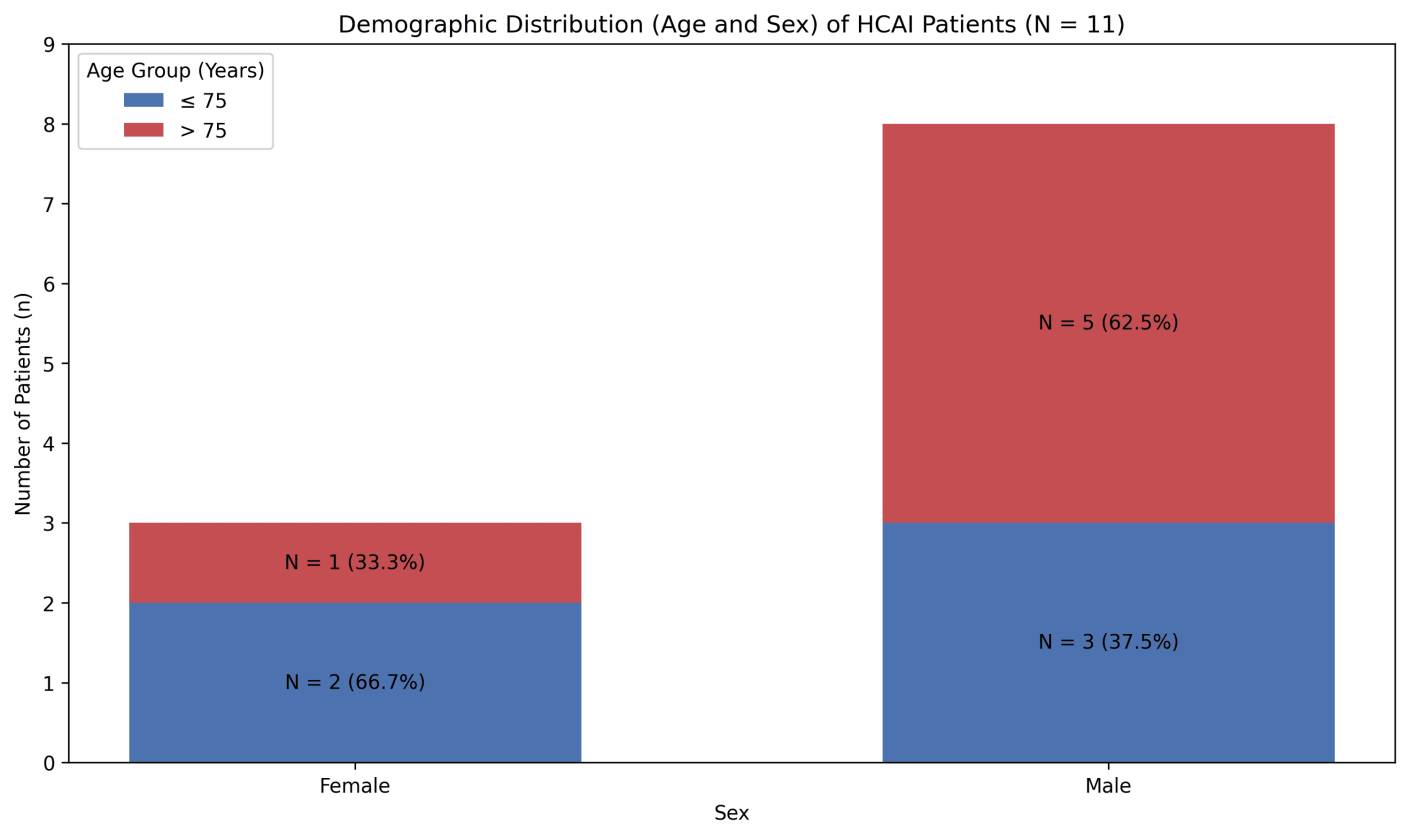
Demographic distribution of study participants: age and sex. HCAI = healthcare-associated infection

Our study showed that 12% (11 patients) of patients who were MOFD developed an HCAI. This was much higher than the rate (6.4% of inpatients suffered HCAI) reported by the HPA [2]. Overall, eight patients were male, of whom five (62.5%) were over the age of 75 years, compared to three female patients, of whom only one (33.3%) patient was over the age of 75 years (Figure 1). One (9%) patient who contracted an HCAI died as a direct result of the HCAI (HCAI listed as cause of death on death certification), and they were in the group of over 75 years old.

**Figure 2:**
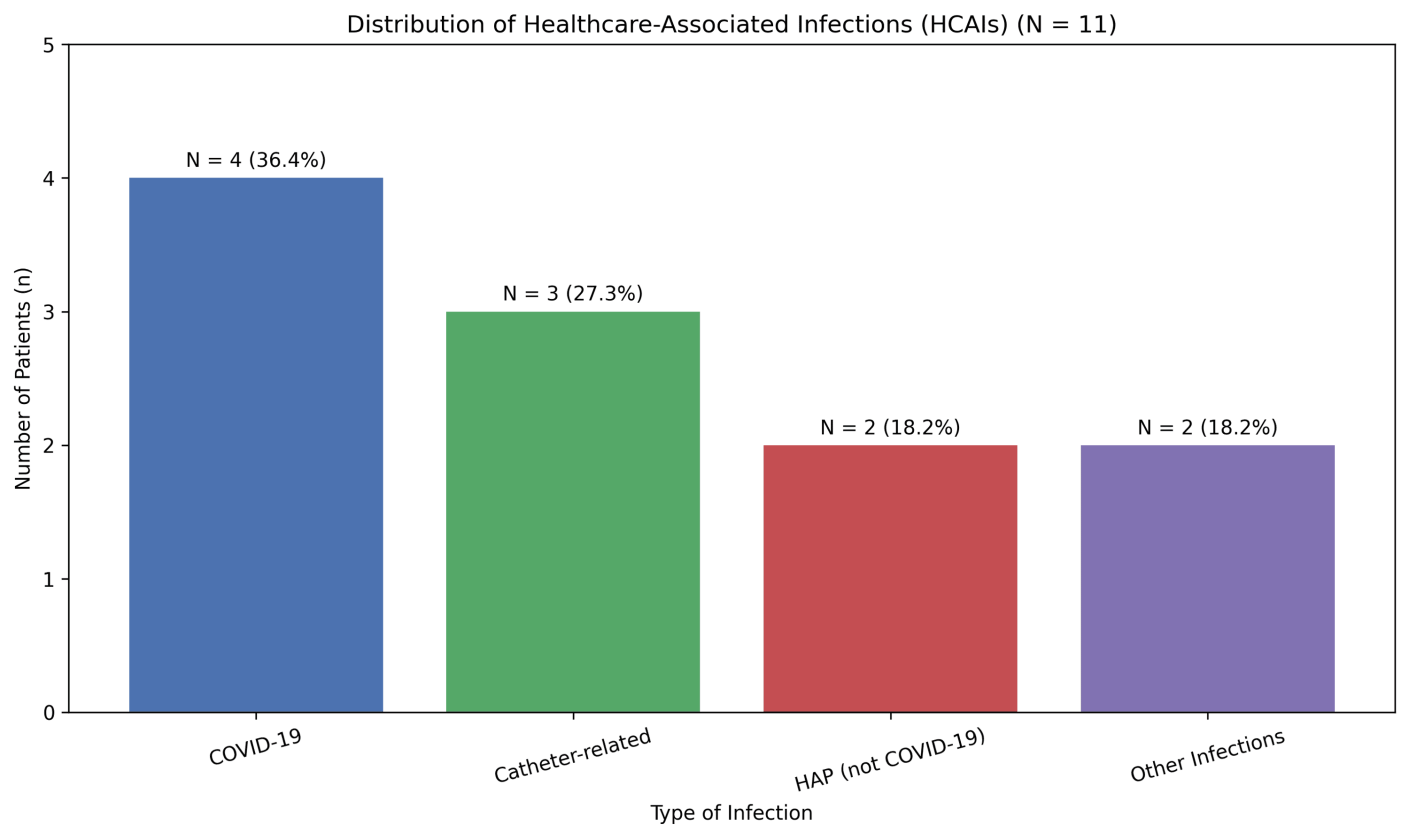
Type and prevalence of HCAI. HCAI = healthcare-associated infection; HAP = hospital-acquired pneumonia

Amongst these patients, COVID-19 (cases confirmed by nucleic acid amplification test, polymerase chain reaction, or rapid antigen testing) was the most prevalent HCAI, affecting four (36.36%) patients, followed by urinary catheter-associated infections in three (27.27%) patients, and hospital-acquired respiratory infections not related to COVID-19 in two (18.18%) patients. Other infections, including norovirus and other respiratory viral illnesses (not causing pneumonia), accounted for the remainder of the infections (Figure 2).

**Figure 3:**
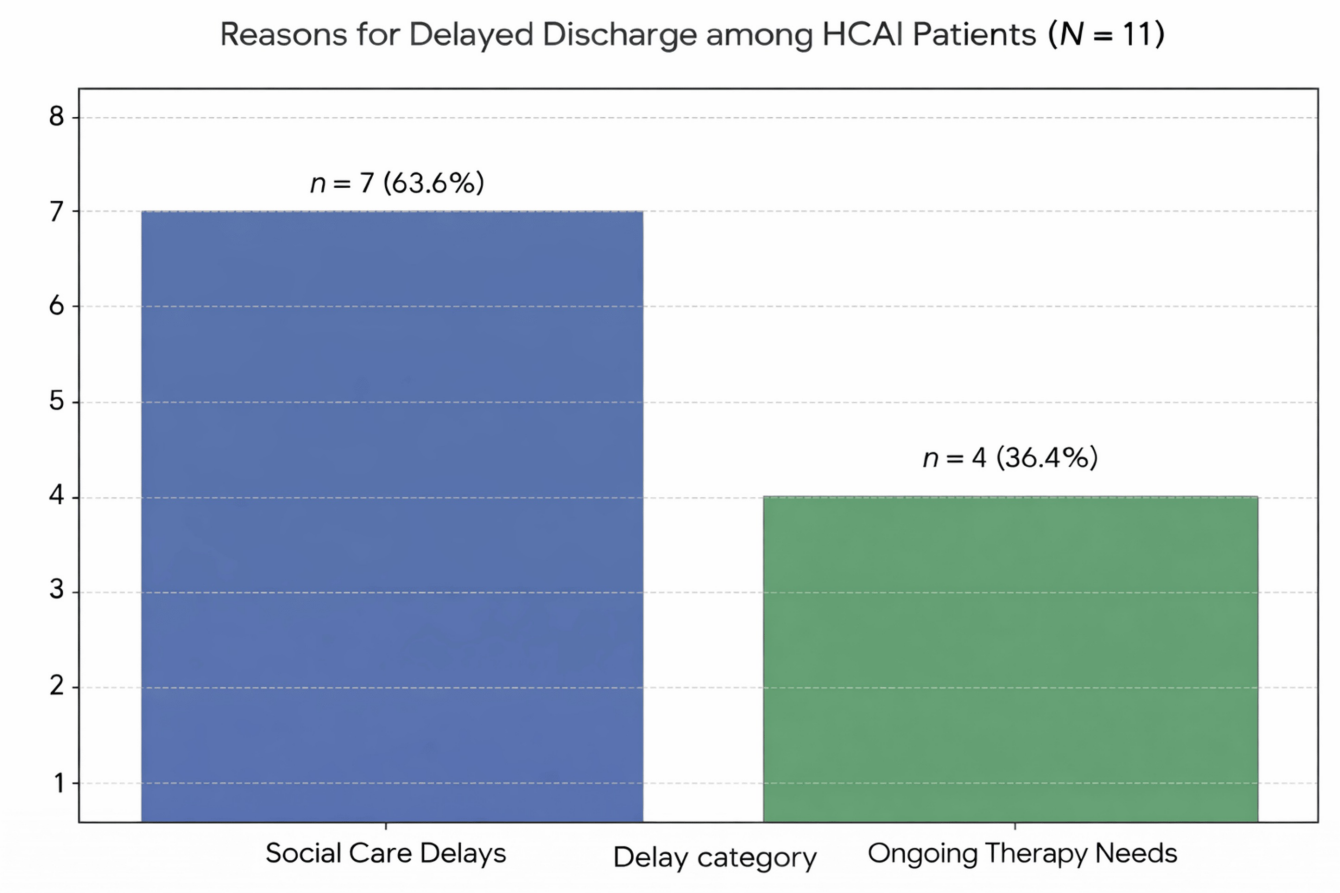
Reasons for delayed discharge amongst HCAI patients. HCAI = healthcare-associated infection

When investigating the reasons for the delay in discharge (Figure 3), the common trends could be divided into the following two categories: delays in arranging social care and ongoing therapy needs (such as requiring further physiotherapy and/or occupational therapy). The majority of patients, 64% (seven patients), had delays in discharge due to delays in arranging social care to facilitate safe discharge. Usually, this was either awaiting a placement in a care home or awaiting social worker allocation. The remainder of these patients had ongoing therapy needs.

## Discussion

HCAIs represent a substantial health and financial burden on both the healthcare system and society. While robust infection prevention measures, such as effective hand hygiene and appropriate isolation protocols, are fundamental to reducing transmission, it is equally important to minimise avoidable exposure by ensuring that medically optimised patients are discharged in a timely manner. Prolonged inpatient stays increase the risk of acquiring infections that may otherwise have been preventable.

The incidence of HCAIs in our cohort was 12%, which is notably higher than figures reported in large population-level surveys. The standard we used for the audit, the 2011 Point Prevalence Survey conducted by Public Health England [2], reported an inpatient HCAI prevalence of 6.4% across acute hospitals in England, roughly half the rate seen in our sample. More recent European surveillance reflects this. The 2022-2023 ECDC Point Prevalence Survey [6] identified an overall adjusted HCAI prevalence of 8.0%, corrected for results of national validation studies. This discrepancy may reflect the vulnerability of patients who remain in hospital beyond the status of medical optimisation, a population typically characterised by advanced age, comorbidity, and prolonged length-of-stay factors (such as requirement for physiotherapy) consistently associated with elevated HCAI risk.

Our findings also align with wider European evidence demonstrating the substantial burden HCAIs pose to healthcare systems. Cassini et al. [7] estimated, using incidence-based disability-adjusted life year (DALY) modelling, that HCAIs account for over 2.5 million cases annually in the EU/EEA, and subsequently correspond to approximately 2.5 million DALYs, resulting in a disease burden comparable to the combined impact of influenza, tuberculosis, and HIV. Their study highlights that the most common HCAIs, namely, respiratory, urinary tract, surgical site, and bloodstream infections, together contribute to more than 90% of the burden of HCAIs. Although our study population is small and local, the distribution of infection types broadly echoes these European trends, particularly the predominance of urinary tract and respiratory infections.

COVID-19 (confirmed by NAAT, PCR, or rapid antigen testing) was the most frequent HCAI in our cohort (36%), reflecting the ongoing challenge of preventing transmission of COVID-19 in general medical wards, particularly among older adults. This pattern is consistent with international analyses reporting high rates of hospital-acquired COVID-19 infection, especially during the endemic phases of the pandemic and ongoing to this day. Iancu et al. [8] identified respiratory infections, led by COVID-19, as a leading category of HCAIs in their multi-centre analysis. They identified *Clostridioides difficile* (32%) as the leading cause, closely followed by COVID-19 (19%). When combining all respiratory infections, including COVID-19, this results in the leading cause of HCAIs. Regarding microbial causes, *Acinetobacter baumannii* was the predominant microbial aetiology of bronchopneumonia, while *Klebsiella pneumoniae* was the leading microbial aetiology of sepsis. *Escherichia coli* was the primary microbial agent causing urinary tract infections, and methicillin-resistant *Staphylococcus aureus* was identified as the main aetiology for wound infections and central catheter infections [8]. As further evidence of the breakdown of HCAIs, the most common types of HCAIs were surgical site infection (23.5%), lower respiratory tract infection (21.6%), and urinary tract infection (19.0%) in German acute care hospitals [9], which is relatively close to our own findings.

A key finding of this study is that delayed discharge, predominantly caused by waits for social care, appears closely linked with increased HCAI risk among the inpatient population. This is strongly supported by the literature. The ECDC PPS 2022-2023 [7] reported that the majority of HCAIs occur after day seven of admission, which highlights length-of-stay as a risk factor. Patients who are medically optimised, yet remain in hospital due to systemic discharge delays (such as awaiting social care), may therefore be at risk of avoidable harm through HCAIs. Our finding that 64% of MOFD patients who developed HCAIs were waiting for social care underscores this system-level delay cause, and aligns with growing concern about ‘delayed transfer of care’ and its clinical consequences.

NHS England (NHSE) data [10] on discharge delays highlight the progress being made to reduce the length of stay in acute hospitals, thereby lowering the risk of HCAIs. This data emphasises that the primary barrier to timely discharge is the delay in arranging social care and securing appropriate post-hospital pathways for patients once they have been deemed medically optimised. Our study supports this conclusion, identifying delays in social care provision as the most significant contributor to prolonged hospital stays among this population.

Recent years have also seen a shift toward admission avoidance models, such as same-day emergency care and specialised outpatient clinics, which have proved effective for a younger and more clinically stable cohort. However, for older or more complex patients, especially those requiring social care input, timely discharge remains a significant challenge.

Despite the important insights provided by our findings, there are several limitations to be acknowledged. First, the retrospective observational design relies on electronic medical record documentation, introducing the potential for incomplete, inconsistent, or inaccurate data. Second, the study was conducted exclusively in general internal medicine wards within a single tertiary centre over a two-month period, which limits generalisability to other settings such as surgical wards, emergency departments, intensive care units, or smaller district general hospitals. Patterns of HCAIs and reasons for discharge delays may vary considerably across these contexts. Third, the sample size was relatively small (n = 93), with only 11 patients developing a HCAI, limiting statistical power and the precision of estimates. Moreover, this study did not take into account the baseline frailty and comorbid status of the patients involved. Additionally, the study did not assess factors such as infection control practices, staff-to-patient ratios, ward occupancy, or crowding, all of which may influence infection risk. Finally, the study did not account for seasonal variation; the data were collected exclusively during December 2023 and January 2024, months typically associated with increased circulation of respiratory viruses, including COVID-19, which may have inflated the observed incidence of certain infections relative to annual trends. Data collection from all seasons would provide more accurate year-round analysis.

Despite these limitations, our findings highlight a population at particular risk of avoidable harm through HCAIs, that is, the MOFD population. Reducing inpatient exposure for medically optimised patients should be a priority in infection-prevention strategies, consistent with NICE guidance [5] on reducing HCAIs. Further prospective work would be advised to quantify the incremental risk associated with each additional inpatient day among MOFD patients and to evaluate whether early-discharge pathways or enhanced community capacity may reduce total HCAI incidence.

## Conclusions

Our study demonstrates that older patients are disproportionately affected by HCAIs, with higher rates of adverse outcomes, including mortality. As the hospital population continues to age and present with greater multi-morbidity, reducing avoidable inpatient exposure is essential to improving patient safety. Strengthening discharge pathways, particularly for medically optimised patients, must therefore be a priority. This includes improving the capacity and accessibility of social care services, as well as expanding community-based rehabilitation and therapy options that allow patients to continue recovery safely outside the acute hospital environment. Investing in these systems would not only reduce the risk of HCAIs but also enhance patient experience, optimise hospital flow, and support more sustainable use of NHS resources. Improving coordination between hospital and community services remains central to achieving better overall outcomes for patients.
